# RETRACTION: Role of Nutritional Support in Nursing Practice for Improving Surgical Site Wound Healing in Patients Post‐Surgery With Risk of Pressure Ulcers

**DOI:** 10.1111/iwj.70585

**Published:** 2025-04-18

## Abstract

**RETRACTION:** ChuY. , KongJ. , XuJ. , HanG. , YuW. , and XuX. , “Role of Nutritional Support in Nursing Practice for Improving Surgical Site Wound Healing in Patients Post‐Surgery With Risk of Pressure Ulcers,” International Wound Journal 21, no. 4 (2024): e14855, 10.1111/iwj.14855.38562093 PMC10985497

The above article, published online on 02 April 2024, in Wiley Online Library (http://onlinelibrary.wiley.com/), has been retracted by agreement between the journal Editor in Chief, Professor Keith Harding; and John Wiley & Sons Ltd. Following an investigation by the publisher, all parties have concluded that this article was accepted solely on the basis of a compromised peer review process. In addition, the investigation found that the authors provided incomplete ethical approval information for the study. The editors have therefore decided to retract the article. The authors did not respond to our notice regarding the retraction.

**RETRACTION:** Y. Chu , J. Kong , J. Xu , G. Han , W. Yu , and X. Xu , “Role of Nutritional Support in Nursing Practice for Improving Surgical Site Wound Healing in Patients Post‐Surgery With Risk of Pressure Ulcers,” International Wound Journal 21, no. 4 (2024): e14855, 10.1111/iwj.14855.38562093 PMC10985497

The above article, published online on 02 April 2024, in Wiley Online Library (http://onlinelibrary.wiley.com/), has been retracted by agreement between the journal Editor in Chief, Professor Keith Harding; and John Wiley & Sons Ltd. Following an investigation by the publisher, all parties have concluded that this article was accepted solely on the basis of a compromised peer review process. In addition, the investigation found that the authors provided incomplete ethical approval information for the study. The editors have therefore decided to retract the article. The authors did not respond to our notice regarding the retraction.

